# ATXN3 promotes prostate cancer progression by stabilizing YAP

**DOI:** 10.1186/s12964-023-01073-9

**Published:** 2023-06-22

**Authors:** Longxiang Wu, Zhenyu Ou, Peihua Liu, Cheng Zhao, Shiyu Tong, Ruizhe Wang, Yangle Li, Junbin Yuan, Minfeng Chen, Benyi Fan, Xiongbing Zu, Yongjie Wang, Jianing Tang

**Affiliations:** 1 Department of Urology, Xiangya Hospital, Central South University, Changsha, China; 2 Department of Burns and Plastic Surgery, Xiangya Hospital, Central South University, Changsha, China; 3 Department of General Surgery, Xiangya Hospital, Central South University, Changsha, China

**Keywords:** Prostate cancer, ATXN3, YAP, Stabilization, Ubiquitination

## Abstract

**Background:**

Prostate cancer (PC) is the most common neoplasm and is the second leading cause of cancer-related deaths in men worldwide. The Hippo tumor suppressor pathway is highly conserved in mammals and plays an important role in carcinogenesis. YAP is one of major key effectors of the Hippo pathway. However, the mechanism supporting abnormal YAP expression in PC remains to be characterized.

**Methods:**

Western blot was used to measure the protein expression of ATXN3 and YAP, while the YAP target genes were measured by real-time PCR. CCK8 assay was used to detect cell viability; transwell invasion assay was used to measure the invasion ability of PC. The xeno-graft tumor model was used for in vivo study. Protein stability assay was used to detect YAP protein degradation. Immuno-precipitation assay was used to detect the interaction domain between YAP and ATXN3. The ubiquitin-based Immuno-precipitation assays were used to detect the specific ubiquitination manner happened on YAP.

**Results:**

In the present study, we identified ATXN3, a DUB enzyme in the ubiquitin-specific proteases family, as a bona fide deubiquitylase of YAP in PC. ATXN3 was shown to interact with, deubiquitylate, and stabilize YAP in a deubiquitylation activity-dependent manner. Depletion of ATXN3 decreased the YAP protein level and the expression of YAP/TEAD target genes in PC, including CTGF, ANKRD1 and CYR61. Further mechanistic study revealed that the Josephin domain of ATXN3 interacted with the WW domain of YAP. ATXN3 stabilized YAP protein via inhibiting K48-specific poly-ubiquitination process on YAP protein. In addition, ATXN3 depletion significantly decreased PC cell proliferation, invasion and stem-like properties. The effects induced by ATXN3 depletion could be rescued by further YAP overexpression.

**Conclusions:**

In general, our findings establish a previously undocumented catalytic role for ATXN3 as a deubiquitinating enzyme of YAP and provides a possible target for the therapy of PC.

Video Abstract

**Supplementary Information:**

The online version contains supplementary material available at 10.1186/s12964-023-01073-9.

## Background

Prostate cancer (PC) is mainly observed as adenocarcinoma of epithelial cell-origin, which is one of the growing public health problems worldwide. Prostate cancer is the most common neoplasm and is the second leading cause of cancer-related deaths in men worldwide. It is estimated that there will be 268,490 newly diagnosed people in 2022, accounting for 27% of all cancer diagnoses in men [1]. There exist numerous treatment options for the localized prostate cancer, surgical removal of the prostate and radiotherapy are the standard treatment options [2, 3]. Despite current developments in the clinical interventions of prostate cancer. The advanced stage of prostate cancer is still a therapeutic challenge. Some patients with primary prostate cancer underwent initial treatments will eventually develop metastatic prostate cancer, which is currently an incurable disease [4, 5]. It is necessary to find out more novel biomarkers for the diagnosis and treatment of prostate cancer.

The Hippo pathway is a newly identified pathway, which plays important roles in organ size regulation and tissue homeostasis. Accumulating studies indicates that dysregulation of Hippo pathway is involved in the initiation and progression of many human cancers [6, 7]. YAP is one of evolutionarily conserved key executors of the Hippo pathway. As a transcriptional co-activator, YAP regulates gene transcription to mediate the biological functions of the Hippo pathway [8]. High activity of YAP enables the cell to escape contact inhibition and anoikis and to support anchorage-independent growth [6]. In addition to the tumor-promoting role of YAP, its activity is also associated with drug resistance. Breast cancer cells with high YAP activity exhibit resistance to drugs such as 5-fluorouracil, taxol, and doxorubicin [9, 10]. The protein stability of YAP is mainly regulated by phosphorylation and ubiquitination [11]. In humans, the kinase cascade MST1/2-Lats1/2 directly phosphorylates YAP, which leads to cytoplasmic retention of phosphorylated YAP and consequentially results in ubiquitination and subsequent proteasomal degradation [12]. It is well known that the ubiquitin–proteasome system (UPS) plays a crucial role in tumorigenesis. The dysregulation of deubiquitinating enzymes (DUB) has been observed in many types of human cancers. It has been previously reported that the deubiquitination enzyme USP9X deubiquitinates and stabilizes YAP in breast cancer cells, thus promoting cancer cell survival [13]. While the deubiquitination and stabilization function of DUB responsible for YAP in prostate cancer is currently unclear. It is important to identify novel regulators in controlling YAP stabilization which can be exploited for potential therapeutic interventions.

In the present study, we screened a DUB siRNA library and identified that a deubiquitinating enzyme, ATXN3, regulates prostate cancer cells proliferation, invasion, and stemness via the Hippo pathway. Mechanistically, ATXN3 deubiquitinates and stabilizes YAP in a proteasome-dependent manner. Furthermore, ATXN3 is overexpressed prostate cancer samples, suggesting that ATXN3 may play a role in the pathogenesis of prostate cancer.

## Methods

### Cell culture

The human prostate cancer cell lines LnCap, C4-2B and human embryonic kidney HEK293T cells purchased from the Procell Life Science&Technology Co,, Ltd (China). All cell lines were authenticated by the cell banks with short tandem repeat analysis. LnCap and C4-2B were culture in RPMI-1640 medium supplemented with 10% fetal bovine serum (FBS, Gibco, Life Technologies, 10,270). HEK293T cells were maintained in Dulbecco’s modified Eagle’s medium (DMEM, 41,965, Life Technologies) supplemented with 10% FBS. All cells were cultured at 37 °C, in 5% CO_2_ humid atmosphere.

### Plasmids and RNA inference

The ATXN3, YAP and mutant plasmids were obtained from Hanbio Biotechnology Co., Ltd. (Shanghai, China). The HA-K6, -K11, -K27, -K29, -K33, -K48, -K63, and -Ub plasmids were acquired from Addgene. Small interfering RNAs (siRNAs) targeting ATXN3 (siRNA-1: 5′-ACAGGAAGGUUAUUCUAUA-3′; 5′- GGACAGAGUUCACAUCCAU-3′) were synthesized by Genepharma (Shanghai, China).

### RNA extraction and qRT-PCR analysis

Hipure total RNA mini kit (Magen, China) was used to isolate the total RNA from the cancer cells. Reverse transcription was performed using the RevertAid First Strand cDNA Synthesis Kit (Thermo, Lithuania) according to the instructions. The SYBR green mix (Toyobo, Japan) and 7500 Fast Real-Time PCR System (Applied Biosystems, Singapore) were used for qRT-PCR analysis. Primers were listed as follows: GAPDH (forward: 5′-ACGGGAAGCTTGTCATCAAT-3′, reverse: 5′-TGGACTCCACGACGTACTCA-3′); YAP (forward: 5′- TAGCCCTGCGTAGCCAGTTA-3′; reverse: 5′- TCATGCTTAGTCCACTGTCTGT-3′); ANKRD1 (forward: 5′- AGAACTGTGCTGGGAAGACG -3′; reverse: 5′- GCCATGCCTTCAAAATGCCA -3′); CTGF (forward: 5′- CTCGCGGCTTACCGACTG -3′; reverse: 5′- GGCTCTGCTTCTCTAGCCTG -3′); CYR61 (forward: 5′- AGCAGCCTGAAAAAGGGCAA-3′; reverse: 5′- AGCCTGTAGAAGGGAAACGC-3′).

### Luciferase assay

The YAP/TEAD luciferase reporter plasmid, Renilla plasmid and ATXN3 siRNA were transfected together into prostate cancer cells. Luciferase activity was detected using the Dual-Luciferase Reporter kit (Promega, Germany).

### Co-immunoprecipitation assay

Cells were washed with pre-chilled phosphate-buffered saline (PBS) and lysed with RIPA extraction reagent (Meilun, China) supplemented with protease inhibitors (Meilun, China). Cell lysates were pre-cleared and incubated with indicated antibody overnight at 4 °C, the antibody associated with the protein complex were then incubated with protein A/G PLUS-Agarose beads for additional 2 h. The beads were washed with PBS three times and boiled at 100 °C for 10 min to reverse crosslinking before SDS-PAGE immunoblotting analysis.

### Protein stability assay

To measure the half-life of YAP, cells were treated with 100 µM protein synthesis inhibitor cycloheximide (Sigma Aldrich) for indicated times. Western blot was performed to measure protein levels.

### In vivo deubiquitination assay

For in vivo deubiquitination assay, HA-Ub, Flag-YAP, Myc-ATXN3 or Myc-ATXN3^C14A^ plasmid were transfected into HEK293T cells for 48 h. Cells were then treated with 10 μM MG132 (MCE) for 6 h. Then cells were washed with pre-chilled phosphate-buffered saline (PBS) and lysed with RIPA extraction reagent. HA-ubiquitinated YAP was isolated using an anti-Flag antibody. The ubiquitination level of YAP was detected by Western blotting with an anti-HA antibody. In prostate cancer cells, HA-Ub plasmid and ATXN3 siRNAs were co-transfected into LnCap cells. HA-ubiquitinated YAP was isolated using an anti-YAP antibody. The ubiquitination level of YAP was detected by Western blotting with an anti-HA antibody.

### Western blot analysis

NP-40 lysis buffer supplemented with protease inhibitors (Meilun, China) was used to extract total protein. BCA Reagent (Thermo Scientific, Rockford, IL, USA) was used to measure the protein concentration. We used an SDS–polyacrylamide gel was to separate the total proteins and transferred proteins to 0.45 μm PVDF membrane (Millipore, USA). The primary antibodies used for western blot analysis are listed as follows: YAP (Proteintech, 66,900–1-Ig), ATXN3 (Proteintech, 13,505–1-AP), GAPDH (Proteintech, 60,004–1-Ig), Myc (Proteintech, 60,003–2-Ig), HA (Proteintech, 51,064–2-AP) antibodies. Signals were detected and visualized using ECL (Meilun, China) and ChemiDocMP imager (Bio-Rad).

### Cell proliferation analysis

For cell proliferation assay, LnCap and C4-2B cells were seeded in 96-well culture plates (2000 cells per well). The proliferation rate of prostate cancer cells was detected using Cell Counting Kit-8 (CCK8) assay at 0 h, 24 h, 48 h, 72 h and 96 h according to the manufacturer's instructions. The absorbance of each well was detected at a wavelength of 450 nm (SpectraMax M5, Molecular Devices, US). For clone formation assay, LnCap and C4-2B cells were seeded into 6-well plates (1000 cells per well) and incubated for 14 days, cells were washed with PBS and stained with 0.5% crystal violet. EdU incorporation assay was performed as we previously reported [14].

### Cell invasion analysis

We performed transwell invasion assay using 8 μm pore polycarbonate membrane transwell plates (Corning, USA). Briefly, 5 × 10^5^ cells were suspended without serum and were cultured in the upper chambers of the transwell plates. The bottom chambers were filled with 600 μl complete medium. After 24 h, the invasion cells were fixed and visualized by crystal violet staining.

### Sphere formation assay

2 × 10^3^ single cells were seeded into 6-well ultra-low attachment culture plates (Corning, USA) in serum-free DMEM/F12 supplemented with 20 ng/ml EGF, B27 (1:50), and 20 ng/ml bFGF. Two weeks later, the spheres were photographed and counted.

### In vivo tumorigenesis assay

Animal experiments were conducted according to the protocols approved by ethnic committee of Xiangya Hospital. 1 × 10^6^ C4-2B cells were resuspended in 100 μl DMEM and injected subcutaneously into the flanks of BALB/c nude mice aged 6 weeks. We used a vernier caliper to measure the tumor sizes and recorded every other day until the end of the experiment.

## Results

### ATXN3 depletion inhibits Hippo signaling pathway activity

**Fig. 1:**
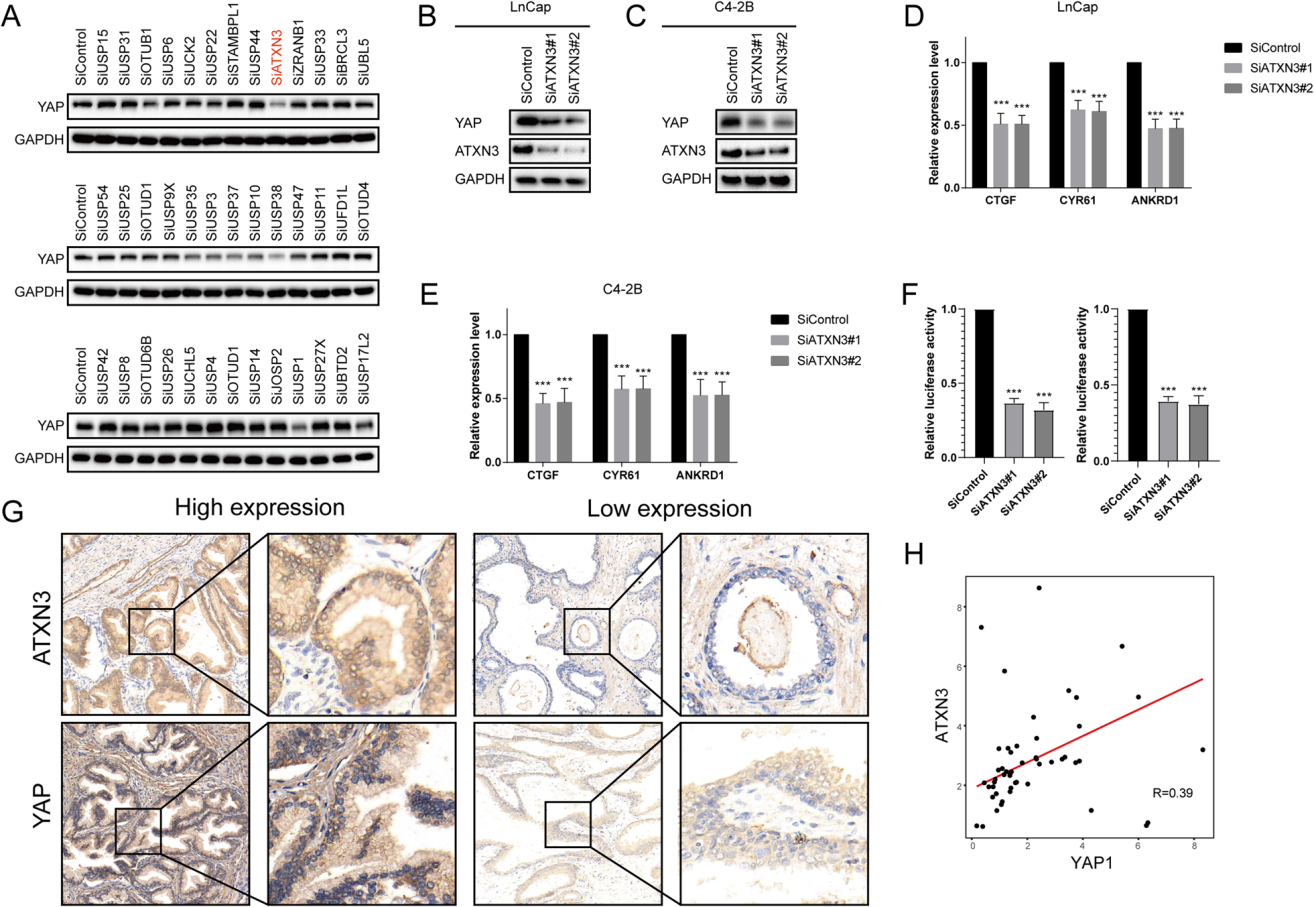
ATXN3 depletion decreases Hippo signaling activity in prostate cancer cells. **A** The siRNAs specific to each deubiquitinating enzyme were transfected into LnCap cells. After 48 h, cells were lysed and the YAP protein level was analyzed by Western blot. **B**, **C** ATXN3 depletion decreased YAP protein level. **D**, **E** ATXN3 depletion decreased YAP target genes using two different siRNA oligos. **F** ATXN3 depletion decreased TOP-luciferase activity. LnCap and C4-2B cells were transfected with SiATXN3 or SiControl together with YAP/TEAD-luciferase reporter plasmid. Luciferase activity was measured 48 h after transfection. **G** ATXN3 and YAP was upregulated in PC samples. **H** ATXN3 correlated YAP in human PC samples. Results shown are representative of 3 independent experiments. Data are represented as mean ± SD of biological triplicates.*P value < 0.05; **P value < 0.01; ***P value < 0.001 by unpaired, 2-tailed Student’s t tests.

To find potential deubiquitinating enzymes that could regulate Hippo signaling pathway in prostate cancer, we screened a DUB siRNA library. Briefly, we transfected four nonoverlapping siRNA mixtures specific for each of the DUBs into prostate cancer cells and found that silencing ATXN3 significantly decreased YAP protein level in LnCap cells (Fig. 1A). We then depleted ATXN3 in LnCap and C4-2B cells with two non-overlapping siRNAs separately to validate the function of ATXN3 in regulating YAP protein level. The results demonstrated that ATXN3 depletion significantly decreased YAP protein level (Fig. 1B, C). In addition, we examined the expression of YAP target genes (CTGF, CYR61 and ANKRD1) and found depletion of ATXN3 dramatically decreased the transcripts of CTGF, CYR61 and ANKRD1 (Fig. 1D, E). To determine if ATXN3 depletion affected YAP/TEAD transcriptional activity, we measured YAP/TEAD-luciferase reporter gene activity followed by ATXN3 depletion. Our results indicated that ATXN3 depletion decreased YAP-luciferase reporter gene activity in LnCap and C4-2B cells (Fig. 1F). IHC analysis indicated that ATXN3 and YAP were both upregulated in prostate cancer samples (Fig. 1G). Besides, we observed a positive association between ATXN3 and YAP protein levels in human prostate cancer samples (Fig. 1H). Taken together, these results indicated that ATXN3 may be a potential regulator of YAP.

### ATXN3 interacts with YAP

**Fig. 2:**
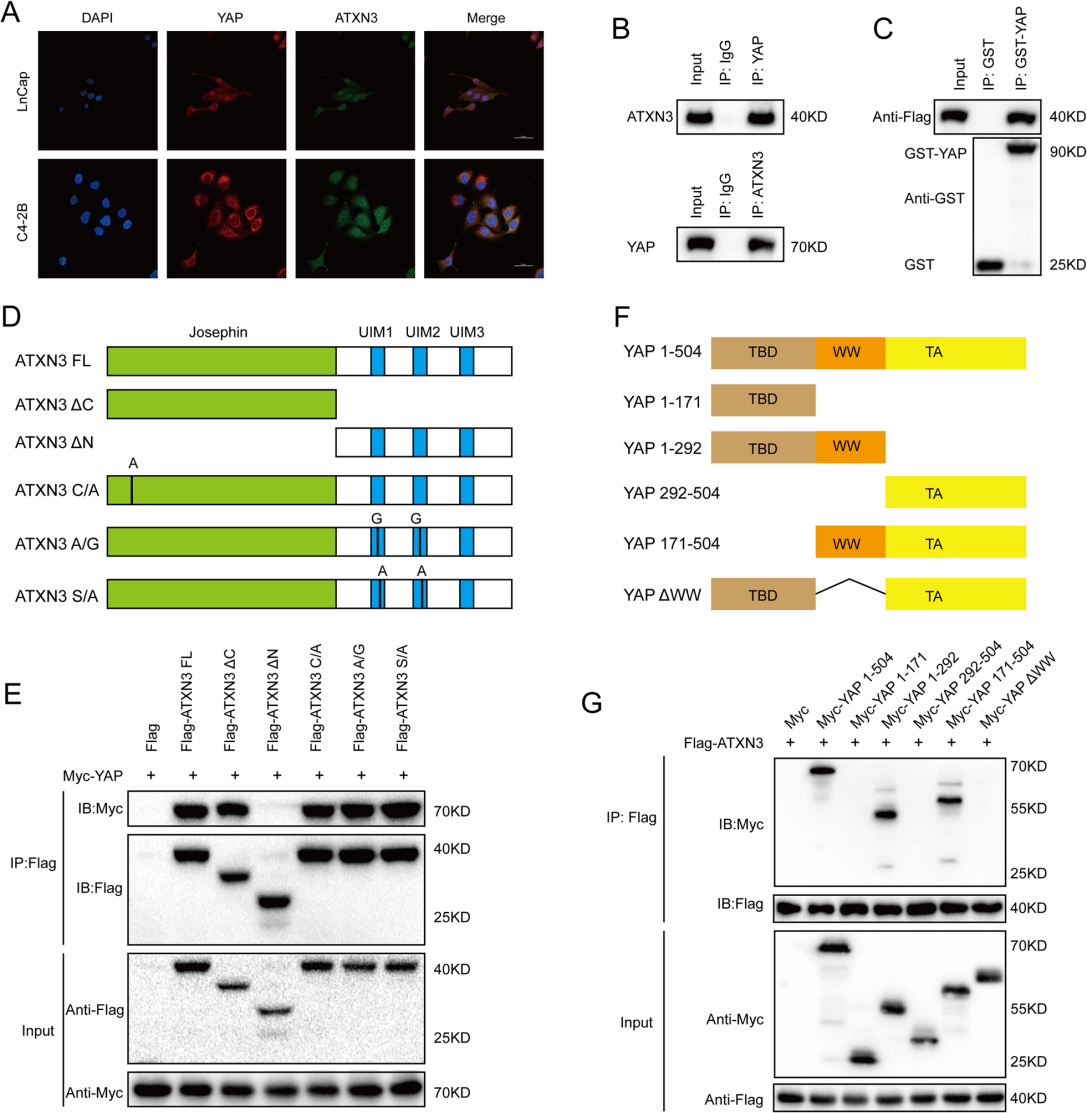
ATXN3 associates with YAP. **A** An immunofluorescence assay demonstrated that ATXN3 and YAP at least partially colocalized in LnCap and C4-2B cells. **B** Co-IP assay reveals association between endogenous ATXN3 and YAP in LnCap cells. LnCap cells were harvested with RIPA lysis buffer. Co-IP was performed using antibody as indicated. **C** LnCap cells transfected with Flag-ATXN3 were lysed and the lysates were incubated with GST-YAP or GST protein. The interacted ATXN3 was detected by western blot. **D** ATXN3 domain structure and deletion mutants used in the study. **E** The Josephin domain of ATXN3 interacted with YAP. HEK293 cells were transfected with 2 µg Myc-YAP together with Flag-ATXN3 full length or mutants. After 24 h, cells were harvested with NP-40 lysis buffer. Co-IP was performed using Myc antibody. The possible interacted ATXN3 domains were detected by Flag antibody. **F**. YAP domain structure and deletion mutants used in the study. **G** The WW domain of YAP interacted with ATXN3. HEK293 cells were transfected with 2 µg Flag-ATXN3 together with Myc-YAP full length or mutants. After 24 h, cells were harvested with NP-40 lysis buffer. Co-IP was performed using Flag antibody. The possible interacted YAP domains were detected by Myc antibody

We then investigated whether ATXN3 co-localizes with YAP in prostate cancer cells, immunofluorescent staining demonstrated that ATXN3 and YAP co-localized in both the cytosol and nucleus of LnCap and C4-2B cells (Fig. 2A). Co-immunoprecipitation (Co-IP) assay indicated the association between ATXN3 and YAP under physiological condition (Fig. 2B). In vitro pulldown assay also detected the direct interaction between YAP and ATXN3 (Fig. 2C). ATXN3 has a structured N-terminal Josephin domain comprising the catalytic site, and an unstructured C-terminal, which contains two or three Ub-interacting motifs (UIMs) depending on the splice isoform (Fig. 2D). We found that full-length ATXN3 could bind to YAP in cells (Fig. 2E). Mutating the active site cysteine 14 of ATXN3 (ATXN3-CA) did not affect its activity binding to YAP, whereas the ΔN deletion mutant of ATXN3, ATXN3-ΔN, lost its interaction with YAP. ATXN3-ΔC and the UIM mutations (ATXN3-SA and ATXN3-AG) showed robust binding to YAP (Fig. 2E). In addition, we observed that the WW domain of YAP was necessary for its binding with ATXN3 (Fig. 2F, G). Taken together, our results demonstrated that the interaction between YAP and ATXN3 is synergistically regulated by the Josephin domain of ATXN3. And the WW domain of YAP is primarily responsible for the interaction with ATXN3.

### ATXN3 stabilizes YAP through the Ub-Proteasome pathway

**Fig. 3:**
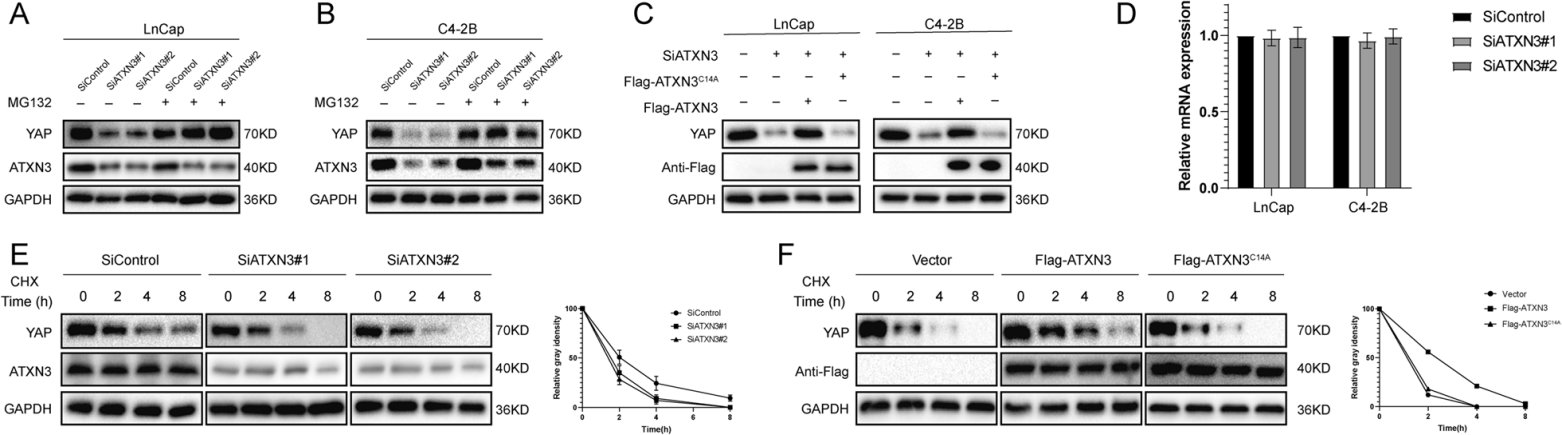
ATXN3 increases YAP stability. **A**, **B** LnCap cells transfected with the indicated siRNA were treated with or without the proteasome inhibitor MG132 (10 µM, 6 h), and then proteins were analyzed. **C** ATXN3 WT or C14A was introduced into LnCap cells together with ATXN3 siRNA. YAP levels were measured. **D** ATXN3 depletion does not affect the mRNA abundance of YAP. **E** LnCap cells transfected with ATXN3 siRNA were treated with cycloheximide (10 µg ml^−1^), and collected at the indicated times for western blot. Quantification of YAP levels relative to GAPDH is shown. **F** Half-life analysis of YAP in LnCap transfected with the indicated plasmids

Since ATXN3 interacted with YAP and ATXN3 depletion decreased YAP levels, we hypothesized that ATXN3 may regulate the turnover of YAP through the Ub-proteasome pathway. Depletion of ATXN3 by two non-overlapping siRNAs separately markedly decreased YAP levels. While in the presence of MG132, YAP protein levels were not affected by ATXN3, indicating that ATXN3 regulated YAP degradation through ubiquitin–proteasome pathway (Fig. 3A, B). Consistently, the decrease of YAP induced by ATXN3 depletion could be reversed by overexpression wild-type (WT) ATXN3, but not its catalytically inactive mutant (ATXN3-CA), suggesting that ATXN3 regulated YAP dependent on its DUB activity (Fig. 3C). Depletion of ATXN3 did not change the mRNA abundance of YAP (Fig. 3D). To prove that ATXN3 affects YAP stability, prostate cancer cells were treated with the protein synthesis inhibitor cycloheximide (CHX). The half-life of YAP was then measured by western blot analysis. As illustrated in Fig. 3E, depletion of ATXN3 markedly decreased the stability of YAP protein. And the half-life of YAP was increased in cells overexpressed ATXN3-WT but not ATXN3-CA (Fig. 3F). These results indicated that ATXN3 stabilizes YAP in cells through the Ub-proteasome pathway.

### ATXN3 deubiquitylates YAP

**Fig. 4:**
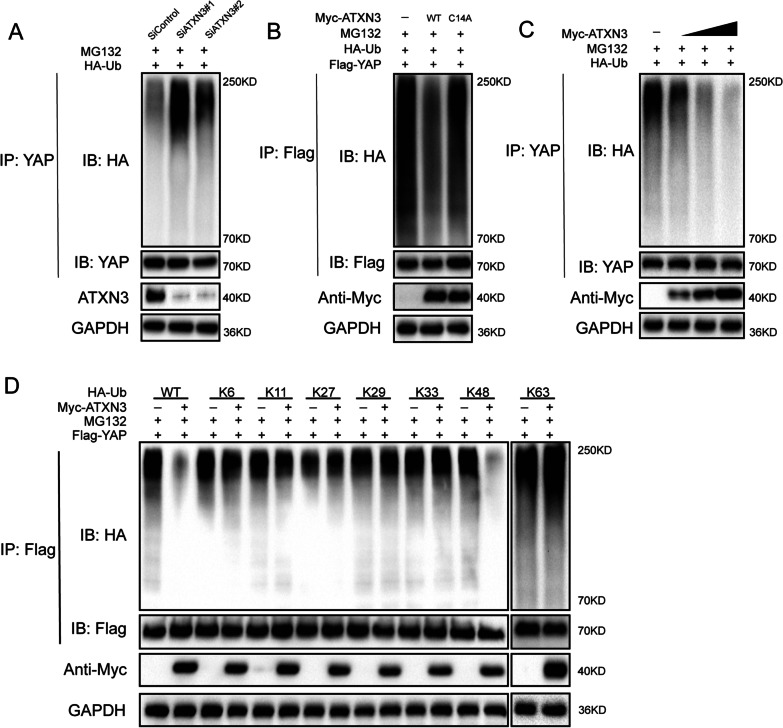
ATXN3 de-polyubiquitylates YAP. **A** LnCap cells transfected with the indicated siRNA were treated with MG132 for 6 h before collection. YAP was immunoprecipitated with anti-YAP and immunoblotted with anti-HA. **B** Immunoblotting was used to detect the ubiquitination of YAP in 293 T cells co-transfected with Flag-YAP, HA-Ubiquitin and Myc-ATXN3 (wild type or C14A). **C** ATXN3 removed the ubiquitin chain of YAP in a dose-dependent manner. **D** HA-WT, K6, K11, K27, K29, K33, K48 or K63 Ub were co-transfected with Flag-YAP and Myc-ATXN3 into HEK293T cells. After treatment with 10 μM MG132 for 6 h, cell lysates were subjected to ubiquitination assay and the ubiquitination level of YAP detected by HA antibody

Since ATXN3 is a deubiquitylase, we went on to examine whether YAP is a substrate of ATXN3. As illustrated in Fig. 4A, depletion of ATXN3 significantly increased the ubiquitination level of YAP. Conversely, ectopic expression of ATXN3-WT, but not ATXN3-CA, markedly decreased YAP ubiquitylation in cells (Fig. 4B). In vivo ubiquitylation assays showed that ATXN3 directly removed the ubiquitin chain of YAP in a dose-dependent manner (Fig. 4C). We also performed ubiquitination assay with a series of mutant ubiquitin (K6, K11, K27, K29, K33, K48, K63) to further investigated which type of ubiquitin chain of YAP was deubiquitylated by ATXN3. It is found that ATXN3 could efficiently remove the K48-linked ubiquitin chain from YAP protein (Fig. 4D). These results suggested that ATXN3 may act as a YAP-directed DUB which deubiquitylated and stabilized YAP.

### ATXN3 regulates prostate cancer progression through YAP

**Fig. 5:**
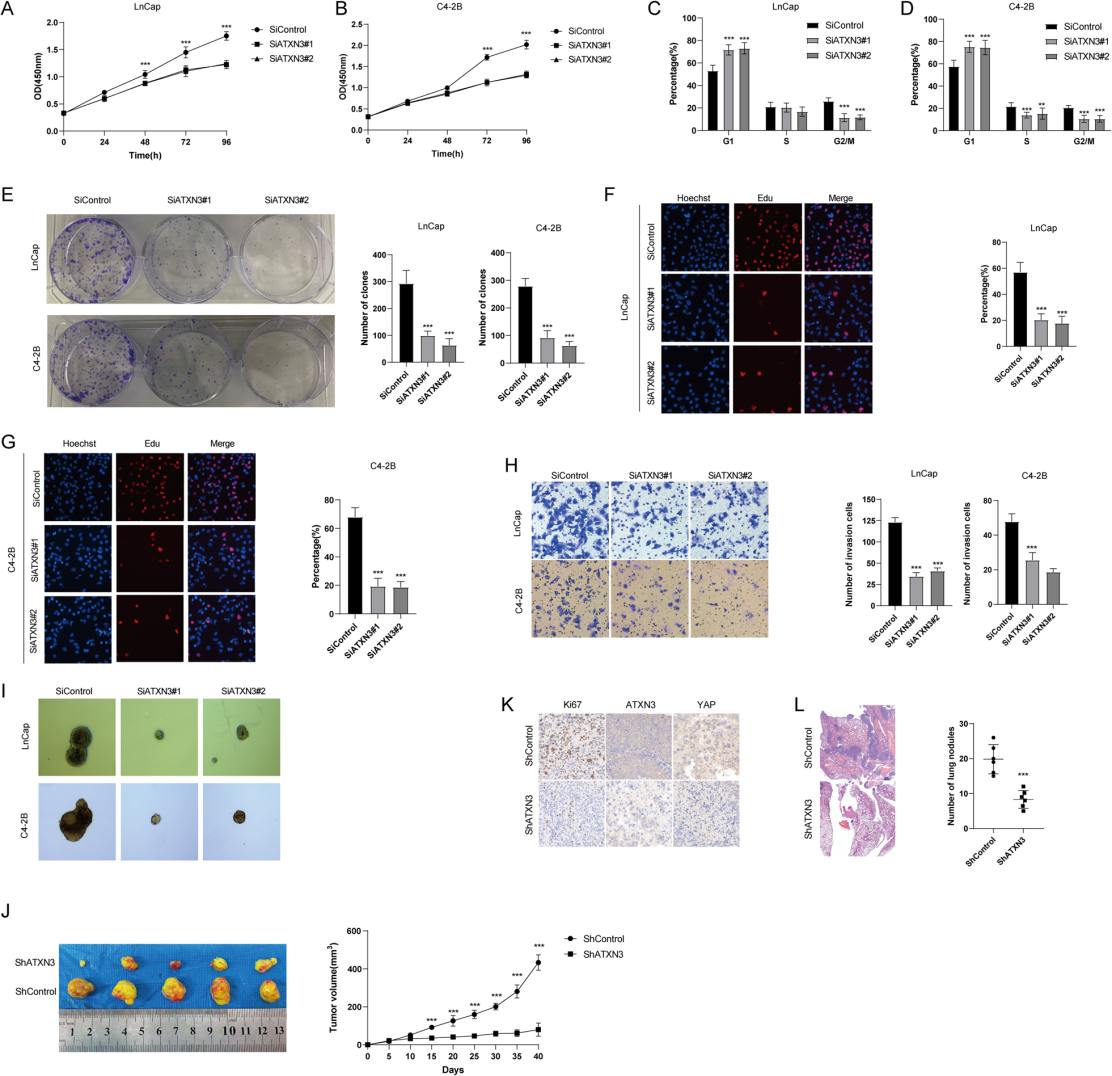
ATXN3 depletion inhibits prostate cancer cell proliferation, invasion stem-like properties. **A, B** ATXN3 depletion inhibited prostate cancer proliferation. **C**, **D** ATXN3 depletion induced G1 cell cycle arrest in prostate cancer cells. **E** ATXN3 depletion decreased clone formation capability of prostate cancer cells. **F**, **G** Representative images of EdU assay of prostate cancer cells. **H** Tranwell invasion assay of prostate cancer cells. **I** ATXN3 depletion decreased sphere formation of HCC cells. **J** ATXN3 depletion inhibits the tumor growth in vivo. C4-2B cells were stably transfected with lentivirus carrying a scrambled shRNA or ATXN3 shRNA. 1 × 10^6^ LnCap cells were injected to the right dorsal flank of each mouse. Tumor sizes were measured every 5 days until the end of the experiment. **K** Representative images of immunohistochemical staining for Ki67, ATXN3 and YAP. (L). ATXN3 depletion suppressed the lung metastasis of ATC in mice. 0.5 × 10^6^ C4-2B cells were intravenously injected into each mouse through the tail vein (n = 6). The lungs were harvested 4 weeks after injection. Results shown are representative of 3 independent experiments. Data are represented as mean ± SD of biological triplicates.*P value < 0.05; **P value < 0.01; ***P value < 0.001

**Fig. 6:**
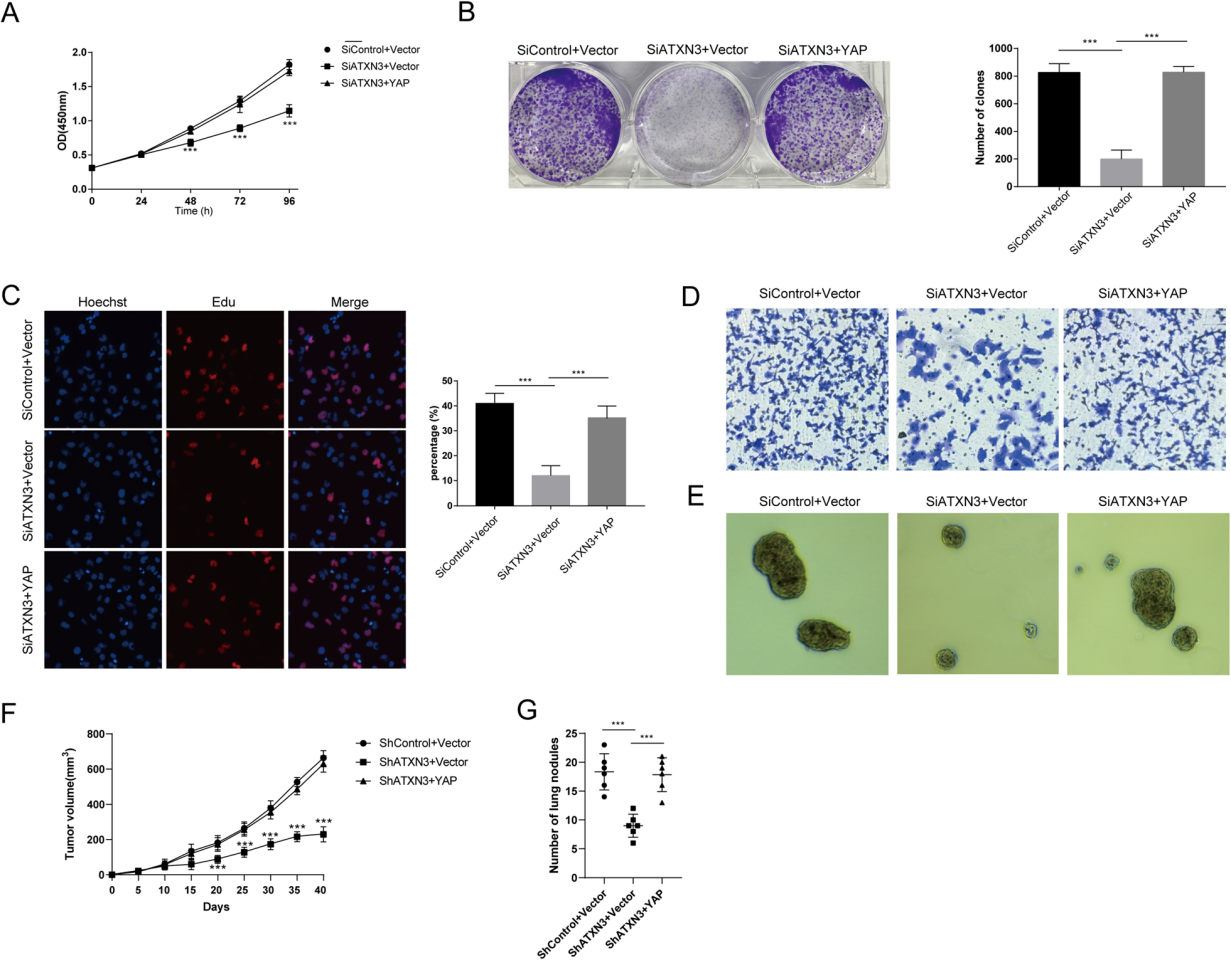
Increased YAP expression reverses the effect induced by ATXN3 depletion. **A** Cell proliferation assay of LnCap. **B** Clone formation assay of LnCap. **C** Representative images of EdU assay of LnCap. **D** Transwell invasion assay of LnCap. **E** Sphere formation assay of LnCap. **F** Overexpression of YAP in ATXN3-knockdown cells partly recovered tumor growth and lung metastasis in vivo. 1 × 10^6^ C4-2B cells transfected with indicated plasmids were injected to the right dorsal flank of each mouse (n = 6). Tumor sizes were measured every 5 days until the end of the experiment. **G** For lung metastasis analysis, 0.5 × 10^6^ C4-2B cells were intravenously injected into each mouse through the tail vein (n = 6). The lungs were harvested 4 weeks after injection. Results shown are representative of 3 independent experiments. Data are represented as mean ± SD of biological triplicates.*P value < 0.05; **P value < 0.01; ***P value < 0.001

We assessed the function of ATXN3 in LnCap and C4-2B cells using two non-overlapping siRNAs separately. The proliferation rate of prostate cancer cells treated with ATXN3 siRNAs was significantly suppressed compared with that in control cells (Fig. 5A, B). ATXN3 depletion significantly inhibited the G1/S phase transition and the colony formation ability of prostate cancer cells (Fig. 5C–E). Consistently, ATXN3 depletion inhibited DNA synthesis as evaluated by Edu incorporation assay (Fig. 5F, G). Transwell invasion assay demonstrated that knockdown of ATXN3 dramatically decreased the invasion capacity of LnCap and C4-2B cells (Fig. 5H). As Hippo signaling is important for maintaining the stemness of tumor cells, we then examined the role of ATXN3 in prostate cancer stemness characteristics. It was found that ATXN3 depletion significantly reduced the oncosphere formation of prostate cancer cells (Fig. 5I). To further confirm the function of ATXN3 in vivo, xenograft experiments demonstrated that depletion of ATXN3 similarly inhibited tumor growth and lung metastasis of prostate cancer (Fig. 5J–L). Since ATXN3 deubiquitinates and stabilizes YAP in prostate cancer cells, we then tested whether ATXN3 regulates these functions via modulating YAP. As shown in Fig. 6A–G, reconstitution of YAP efficiently rescued the suppressive effects induced by ATXN3 depletion. Our results suggested that ATXN3 promotes prostate cancer progression by regulating YAP.

## Discussion

Prostate cancer is one of the most common malignant tumors in males. In the past decades, the diagnosis rates of prostate cancer have dramatically increased since the introduction of prostate-specific antigen (PSA) testing. And with the improvement in the understanding of the molecular mechanisms of prostate carcinogenesis, the mortality of prostate cancer has fallen [15]. Although PSA has been used as an early prostate cancer biomarker, it cannot distinguish between indolent and aggressive prostate cancer [16]. Approximately 10% of prostate cancer presents as lethal prostate cancer, and treating locally advanced or metastatic prostate cancer is still a worldwide medical challenge [17]. Ubiquitination is an important posttranslational modification, which is a central component of the cellular protein-degradation machinery and essential for cellular homeostasis [18]. Ubiquitination involves the sequential transfer of an ubiquitin molecule mediated by three enzymes: ubiquitin-activating enzyme (E1), an ubiquitin conjugating enzyme (E2) and an ubiquitin ligase (E3) [19]. The E3 ubiquitin ligases selectively mediate the ubiquitin conjugation of substrates, which could be reversed by deubiquitinating enzymes [20]. Dysregulation of E3 ligases or deubiquitinating enzymes is frequently observed in various human cancers. However, the potential roles of DUBs in prostate cancer are not well investigated.

YAP activity plays a crucial role in regulating tumorigenesis. Tight regulation of YAP is important for maintaining normal cellular functions. It has been shown that YAP can be regulated at the level of protein stability, which occurs predominantly through the Ub-mediated proteasomal degradation. For instance, Fbxw7 regulates YAP protein stability by targeting YAP for ubiquitination and proteasomal degradation in hepatocellular carcinoma [21]; SHARPIN and RNF187 promote YAP degradation via inducing YAP K48-dependent poly-ubiquitination [22, 23]. Deubiquitinating enzymes (DUB) also regulate the stability of YAP protein in human cancers. In breast cancer, USP9X deubiquitinates and stabilizes YAP to promote breast cancer cell proliferation and chemoresistance to therapeutic drugs [13]. USP47 functions as a DUB for YAP in colorectal cancer, USP47 elevation leads to the stabilization of YAP and promotes colorectal cancer cell proliferation [11]. USP10 directly interacts with and stabilizes YAP/TAZ by reverting their proteolytic ubiquitination. Depletion of USP10 enhances polyubiquitination of YAP/TAZ, promotes their proteasomal degradation, and ultimately arrests the proliferation of hepatocellular carcinoma [24]. OUTB1 interacts with YAP protein via its OTU domain (Ovarian tumor domain) and deubiquitinates YAP at several lysine sites (K90, K280, K343, K494 and K497), which subsequently inhibits YAP degradation [25]. USP22 interacts with and deubiquitinates YAP to prevent YAP turnover [26]. While the DUB responsible for YAP deubiquitination and stabilization in prostate cancer remain elusive. To further systematically evaluate the impact of the individual members of the DUB family in YAP deubiquitination and tumor progression, we have screened a DUB siRNA library and conducted unbiased siRNA screening by monitoring the levels of YAP and identified several candidate DUBs. In addition to ATXN3, we also noticed a few other DUBs with potential effect on Hippo signaling, including USP10 and OTUB1. However, siUSP47, siUSP22 or siUSP9X imposed minimal effect on YAP in our screening systems. The seemingly contradicting results may arise from the variation of cellular context in different cell lines.

ATXN3 is a DUB belonging to the MJD family, which is one of the five main cysteine deubiquitinating enzyme families. It is highly conserved and ubiquitously expressed in cells throughout the body [27]. In ATXN3 KO mice, the absence of ATXN3 results in an increase of total ubiquitinated protein levels [28], whereas overexpression of ATXN3 in HEK293 cells leads to dramatically reduced cellular protein ubiquitination [29]. Recent studies have reported numerous substrates of ATXN3, indicating that ATXN3 is a ubiquitous deubiquitinating enzyme regulating the ubiquitination and stabilization of various proteins [30]. ATXN3 binds to p53, and further deubiquitinates, stabilizes p53 to regulate the functions of p53 in transactivation and apoptosis [31]. ATXN3 could interact with Chk1 and protect it from degradation, thereby promoting DNA repair and checkpoint signaling [32]. Besides, ATXN3 positively regulates type I IFN antiviral response by deubiquitinating and stabilizing HDAC3 [33]. ATXN3 is also involved in gastric and breast cancer. It acts as either a tumor suppressor or an oncogene. The expression of ATXN3 protein was decreased in the gastric cancer compared to noncancerous gastric tissue, and associated with tumor size, histologic differentiation, and Lauren classification [34]. In breast cancer, high expression of both ATXN3 is correlated with a poor prognosis. ATXN3 promotes breast cancer metastasis by stabilizing KLF4 through deubiquitination [35]. To systematically evaluate the impact of the individual members of the DUB family in regulating YAP deubiquitination and promoting tumor progression, we have screened a DUB siRNA library and conducted unbiased siRNA screening by monitoring the levels of YAP. We identified ATXN3 as a deubiquitinating enzyme that stabilizes YAP in prostate cancer. First, ATXN3 and YAP interacted with each other. Co-IP analysis identified the interaction between ATXN3 and YAP. We found that the Josephin domain of ATXN3 is essential for the direct binding of ATXN3 to both YAP. ATXN3-SA and ATXN3-AG showed robust binding to native while compromised binding to ubiquitinated YAP, suggesting that the UIMs in the C-terminal are not required for its binding with YAP. Therefore, the Josephin regulates the interaction between ATXN3 and YAP. Second, ATXN3 decreased YAP polyubiquitination and promotes YAP protein stabilization in a manner that depending on its DUB activity. Deletion of ATXN3 markedly reduced the protein abundance of YAP, and the reduced YAP protein abundance could be restored by ectopic expression of ATXN3-WT, but not its catalytically inactive mutant ATXN3^C14A^.Under the treatment of the proteasome inhibitor MG132, ATXN3 deletion could not further affect YAP protein level. ATXN3 depletion decreased the half-life time of YAP protein. Ectopic expression of ATXN3-WT, but not ATXN3^C14A^, significantly inhibited the ubiquitilation of YAP. In vivo deubiquitylation assays demonstrated that the ubiquitin chain on YAP could be removed by ATXN3 in a dose-dependent manner. To further find out which type of ubiquitin chain on YAP was removed by ATXN3, we performed ubiquitination assay using a series of ubiquitin mutants, including K6, K11, K27, K29, K33, K48 and K63. We observed that ATXN3 significantly decreased K48-linked polyubiquitination from YAP. As polyubiquitination through K48 of Ub generally results in proteasomal degradation [36, 37], ATXN3 may maintain the stability of YAP by removing the K48-linked ubiquitin chain from YAP protein. Finally, ATXN3 could promote tumor proliferation, invasion and stem-like properties of prostate cancer through YAP. Knockdown of ATXN3 significantly inhibited tumor proliferation, invasion and stem-like properties. In addition, the restoration of YAP expression abolished the effects induced by ATXN3 depletion. These results demonstrated that ATXN3 promoted prostate cancer progression through increasing the stability of YAP.

## Conclusions

In conclusion, our present study demonstrated that ATXN3 functions as a DUB responsible for YAP that promotes tumor growth, invasion, tumor stem-like properties of prostate cancer through stabilizing YAP protein. ATXN3 interacted with YAP protein and enhanced its stability via removing the K48-linked ubiquitin chain from YAP. Our study reveals a new regulation mechanism of YAP and indicating that ATXN3 might be further developed into a potential therapeutic target in prostate cancer.

## Data Availability

Data sharing is not applicable to this article as no datasets were generated or analyzed during the current study.
